# Inhibition of TRIF-Dependent Inflammation Decelerates Afterload-Induced Myocardial Remodeling

**DOI:** 10.3390/biomedicines10102636

**Published:** 2022-10-19

**Authors:** Stephanie I. Bettink, Jan-Christian Reil, Andrey Kazakov, Christina Körbel, Dominic Millenaar, Ulrich Laufs, Bruno Scheller, Michael Böhm, Stephan H. Schirmer

**Affiliations:** 1 Internal Medicine III, Saarland University, 66421 Homburg, Germany; 2 Medical Clinic II, University Hospital Schleswig-Holstein, 23538 Lübeck, Germany; 3 Institute for Clinical & Experimental Surgery, Saarland University, 66421 Homburg/Saar, Germany; 4 Department of Cardiology, Leipzig University, 04103 Leipzig, Germany

**Keywords:** TRIF, fibrosis, hypertrophy, inflammation, afterload

## Abstract

Pressure-overload-induced cardiac hypertrophy represents one cause of the development of heart failure. The aim of this study is to characterize the influence of the TIR-domain-containing adapter-inducing interferon-β (TRIF) during afterload-induced myocardial remodeling. After trans-aortic constriction (TAC), cardiac pressure overload leads to an early increase in MyD88- (Myeloid differentiation primary response gene 88) and TRIF-dependent cytokines. The maximum cytokine expression appeared within the first week and decreased to its control level within five weeks. While cardiomyocyte hypertrophy was comparable, the myocardial accumulation of the inflammatory cells was lower in TRIF^−/−^mice. At d7, TRIF deficiency reduced transcription factors and TRIF-dependent cytokines. Through the modulation of the TGF-β-signaling pathway and anti-fibrotic microRNAs, TRIF was involved in the development of interstitial fibrosis. The absence of TRIF was associated with a decreased expression of proapoptotic proteins. In echocardiography and working heart analyses, TRIF deficiency slowed left-ventricular wall thickening, myocardial hypertrophy, and reduces the ejection fraction. In summary, TRIF is an important adapter protein for the release of inflammatory cytokines and the accumulation of inflammatory cells in the early stage of maladaptive cardiac remodeling. TRIF is involved in the development of cardiac fibrosis by modulating inflammatory and fibrotic signal transduction pathways.

## 1. Introduction

Increased afterload, as imposed by arterial hypertension or aortic stenosis, results in pathological myocardial hypertrophy, which has to be differentiated from physiological hypertrophy in pregnancy or exercise [1,2] and is associated with incident heart failure [3]. Ameliorating afterload-induced cardiac remodeling is a primary target for therapeutic approaches in preventing hypertension-induced end-organ damage [4].

During maladaptive myocardial remodeling, the continued loss of cardiomyocytes and the replacement by interstitial fibrosis [5] leads to the deterioration of left-ventricular function. At the molecular level, pressure overload can induce myocardial inflammation with the overexpression of pro-inflammatory cytokines and leukocyte infiltration into the myocardium.

Cardiac remodeling is triggered by cardiac injuries, cytokine secretions, and enhanced autonomic activity [6]. Various observational studies in animals and patients have indicated a relationship between the presence of pro-inflammatory molecules and pressure-overload-induced hypertrophy and fibrosis [7,8,9]. Recently, a prospective clinical investigation showed that interleukin (IL)-1b inhibition reduced heart failure in a patient population with previous myocardial infarction and elevated inflammatory markers [10]. Inflammatory immune responses are initiated via Toll-like receptors (TLR), recognizing pathological molecules, and intracellular signaling processed by MyD88 (Myeloid differentiation primary response 88) or the TRIF (TIR-domain-containing adapter-inducing interferon-β)-dependent pathway. While MyD88 is known as a universal adapter protein used by almost all TLRs (except for TLR 3), TRIF mediates only the TLR3- and TLR4-dependent signaling cascade. The stimulation of TLR-3/TRIF signaling activates transcriptional factors and interferon regulator factor 3/7 and subsequently results in the production of various cytokines [11]. In the heart, TLR3/TRIF signaling is known for its critical role in the host’s innate immune response against virus-induced myocarditis [12,13,14]. TLR4/TRIF plays a role in cardiac remodeling after myocardial infection and cardiac hypertrophy [15,16,17].

We hypothesized that TLR3/4-TRIF inflammatory signaling is involved in cardiac remodeling processes in afterload-induced myocardial hypertrophy. In this study, we determined the time of maximal cytokine expression in TRIF knockout and wild-type mice after trans-aortic constriction (TAC). Furthermore, we focused on inflammatory cell infiltration and fibrosis at the time-point of maximal cytokine expression (day 7).

## 2. Materials and Methods

### 2.1. Animals and Transaortic Constriction

The study was approved by the animal Ethics Committee of Saarland University des and conformed to the Guide for the Care and Use of Laboratory Animals published by the US National Institutes of Health (NIH Pub. No. 85–23, revised 1996).

Age- and sex-matched TRIF-deficient mice (Ticam1Lps2/J) [18] (The Jackson Laboratory) and wild-type C57BL/6J mice (Charles River) were housed under standard conditions. Transverse aortic constriction (TAC) in the mice was performed as established in our laboratory [19]: After anesthetization (ketamine, 100 mg/kg body weight, i.p. and xylazine, 10 mg/kg, i.p.), orotracheal intubation and connection of the tube to a volume cycled rodent ventilator (Harvard Apparatus) on supplemental oxygen (tidal volume of 0.2 mL and respiratory rate of 110 per min), the chest cavity was entered in the second intercostal space at the left upper sternal border through a small incision. Aortic constriction was carried out by looping a 7-0 nylon suture ligature against a 27-gauge needle to yield a restriction of 360 μm in diameter and a transverse aortic constriction of 65–70%. A sham operation was performed on the control mice group. The animals were sacrificed using an i.p. injection of ketamine (1 g/kg body weight) and xylazine (10 mg/kg) after 7 and 35 days of intervention, respectively. The hearts were immediately excised and divided in half. One piece of the heart was rapidly frozen in liquid nitrogen and stored at 80 °C, and the other piece was fixed in 4% PBS-buffered formalin for paraffin embedding. Alternatively, to determine the heart weight, the excised hearts were dried at 60 °C for two days. The spleens were stored in cold PBS for lymphocyte isolation, and venous blood was collected with EDTA.

### 2.2. Echocardiography

The mice were anesthetized with 1.5% isoflurane and imaged using the High-Resolution in vivo Micro-Imaging System Vevo^®^770 (VisualSonics, Toronto, ON, Canada) with the RMV-707B transducer (Real-Time Micro Visualization mouse cardiac, 30 MHz).

In parasternal longitudinal axis projection after the acquisition of the apex and the aortic root in one line, two-dimensional (B-mode) images were taken. At the end diastole and end systole, apical to the papillary muscle, one-dimensional M-mode measurements were taken in the same setting. The left ventricular cavity sizes and wall thicknesses were observed and averaged in this projection for three heart cycles. The interventricular septum (IVSd/s [mm], diastolic/systolic), the left ventricular internal diameter at end-diastole (LVIDd/LVEDD [mm]) and end-systole (LVIDs/LVESD [mm]) and the left ventricular posterior wall (LVPWd/s [mm]; diastolic/systolic) were recorded. In the short-axis projection, the standard measurement of the left ventricular anterior wall (LVAWd/s [mm]; diastolic/systolic) was also performed at three heart cycles. The data were collected and analyzed with the VisualSonics Vevo^®^770 Version 2.3.0 (Visualsonics Inc., Toronto, ON, Canada) Software. The following functional parameters were calculated from the M-mode measurements: Left ventricular mass (LV mass [g]) and left ventricular end-diastolic volume (LVED vol [μL]), as well as the left ventricular shortening fraction (Fractional shortening, FS [%]); see Figure S1 in the Supplementary Materials.

### 2.3. Langendorff-Working Heart Preparation

The studies on the isolated hearts were carried out as previously described [20]. Briefly, the mice were euthanized after an intraperitoneal injection of heparin (1000 IU) and an undiluted Ketavet^®^-Rompun^®^ anesthetic mixture (with a ratio of 2:1, 0.3 mL per 20 g body weight). Immediately after death, the heart was excised and transferred into an ice-cold cardioplegic solution. After the removal of the pericardium, lung remains, and trachea, the aorta was cannulated with an 18 g metal cannula and perfused in the Working Heart apparatus with Krebs–Henseleit buffer and a perfusion pressure of 60 mmHG. After the sufficient perfusion of the heart in the Langendorff mode, a 16 g steel cannula was positioned in the left atrium via the pulmonary vein. As part of the Working Heart preparation, the cannula was then connected to a column filled with Krebs–Henseleit buffer and heated to 38 °C (preload: 10 mmHg, afterload: 60 mmHg). Two platinum electrodes embedded in polyester resin were attached to the right atrium, stimulating the heart rate to approximately 400 beats per minute (bpm).

The left ventricular systolic and diastolic functions were measured using a pressure–volume catheter (Millar 1.4 F SPR-835, Millar, Houston, TX, USA). The catheter was inserted into the left ventricle with a 22 ¼ g needle after puncturing the apex. The end-diastolic pressure–volume ratio (ESPVR), and the associated gradient of EES (end-systolic ventricular elasticity), were assessed by increasing the afterload from 60 mmHg to 100 mmHg. The preload was left constant at 10 mmHG and the heart rate at 400 bpm. Finally, the wall thickness was determined by adding 5 µL of hypertonic saline solution (5%) via the cannula into the left atrium (transient change in the conductivity of the Krebs–Henseleit buffer in the left ventricular cavity).

### 2.4. Cell Isolation and Culture

Neonatal rat cardiomyocytes and cardiac fibroblasts were isolated from 5-day-old neonatal Sprague Dawley rat hearts (Charles River) of mixed sex, as described [19]. Briefly, after a few hours in culture, the cardiomyocytes showed regular spontaneous contractions and were utilized for experiments after 3–5 days. The fibroblasts were separated by adhesion. Neonatal cardiac fibroblasts were grown in Dulbecco’s modified Eagle’s medium (Invitrogen) with 10% (*v*/*v*) fetal calf serum, gentamicin (0.08 mg/mL), and penicillin (100 IU/mL). The cells were used for the experiments when sub-confluent in passage 3. HUVECS were grown in Endothelial-Cell-Growth medium (Promocell, Heidelberg, Germany) and used in passages 3 to 6.

### 2.5. siRNA-Transfection

Neonatal rat cardiomyocytes, neonatal cardiac fibroblasts, or HUVECs were transfected with 15 nM TRIF-siRNA or negative siRNA using the HiPerfect Transfection Reagent (Qiagen, Germantown, MD, USA). The cells were harvested for 48 h and then isolated for protein expression and cell proliferation tests.

### 2.6. Histology

The paraffin-embedded LV heart tissues of the transverse cross-sections (3 µm) were stained with hematoxylin/eosin for the cardiomyocyte area. Several photographs were taken to cover the whole cross-section of the embedded heart. The pictures were magnified electronically, and the cross-sectional area of 100 cells, with nuclei approximately in the center of the area, was microscopically determined with the LUCIA G© Software (Nikon, Tokyo, Japan) at 40× magnification.

Picrosirius-red was performed for the analysis of fibrosis. The collagen content of the cross-section was determined by marking the red areas via LUCIA G© Software (Nikon, Tokyo, Japan). The collage content [%] = area of collagen/area of the cross-section.

### 2.7. Hydroxoproline Assay

The hydroxyproline content was assessed using QuickZyme Hydroxyproline Assays (QuickZyme, Leiden, The Netherlands). After 100 mg of tissue was hydrolyzed, hydroxyproline was determined via the ELISA technique. The content was calculated via a standard curve.

### 2.8. Immunohistochemistry

Immunofluorescence staining was performed for the detection of cardiomyocytes (α sarcomeric actin, and Dianova), macrophages (F4/80, abcam, Waltham, MA, USA), and T-lymphocytes (CD3, abcam) on paraffin-embedded LV heart tissues of the transverse cross-sections (3 µm). After heat-mediated antigen retrieval with citraconic anhydride solution, incubation with the first antibody was performed by overnight incubation at 4 °C, additional at 37 °C for 1 h, and subsequently with the appropriate secondary antibody at 37 °C for 1 h. In 15 areas at 100× magnification, positive cells were counted against all of the DAPI-positive cardiomyocytes.

### 2.9. Detection of Apoptosis

The apoptotic cells were stained on slides (cross-sections of the heart) with the ApopTag^®^ Peroxidase In Situ OligoLigation (ISOL) Apoptosis Detection Kit, Millipore. The ratio of apoptotic and vital cells in 10 areas was determined for quantification.

### 2.10. RNA Extraction and Real-Time PCR for Gene Expression

The RNA from the frozen heart tissue was extracted using the peqGOLD RNAPure solution according to the manufacturer’s instructions (Peqlab Biotechnologie). RNAs from the spleen-derived macrophages and from the cultured cells were isolated with an absolute RNA Microprep Kit (Stratagene, La Jolla, CA, USA). cDNA synthesis was performed with a high-capacity cDNA reverse-transcription kit (Life Technologies, Carlsbad, CA, USA). The quantitative gene expression was measured using the TaqMan system with KAPA SYBR FAST Universal Sybr Green (Peqlab Biotechnologie) and custom-made primers. The heart tissue and macrophages were examined for the expression of different cytokines (See Supplementary Materials, Table S1) and appropriate controls (18s/HPRT). The gene expression was determined as an x-fold difference after normalizing to the controls (WT-TAC).

The detection of microRNA miR29 and miR146a was performed from isolated RNA using the TaqMan^®^ MicroRNA Assay (Life Technologies).

### 2.11. Western Blot Analysis

The protein extracts from the frozen heart tissues and the si-transfected cells were separated by a SDS PAGE (12%) and transferred to nitrocellulose membranes (Bio-Rad, Hercules, CA, USA), followed by the blocking of the non-specific protein with 5% dried milk for 1 h. The blocked membranes were incubated overnight at 4 °C with the primary antibody against IRF3 (cell signaling), TGFβ (Sigma, Burlington, MA, USA), TGFβ-RI, TGFβ-RII, p53, p16, Bax and BCL, and GAPDH (all Santa Cruz). The detection was performed using the suitable horseradish peroxidase-conjugated secondary antibody for 1 h at room temperature in 5% dried milk. The plots were quantified by densitometry and normalized against GAPDH.

### 2.12. Cell Proliferation ELISA

Neonatal rat cardiomyocytes, neonatal cardiac fibroblasts, or HUVECs were seeded in 96-well cell-culture plates, and si-transfection was performed. After 48 h, the cells were used in the Cell Proliferation ELISA, BrdU (Roche, Basel, Switzerland), according to the manufacturer’s instructions.

### 2.13. Statistical Analysis

The results were expressed as mean + SEM. A statistical comparison between the groups was evaluated using one-way ANOVA and additional Bonferroni’s multi-comparison test. Values of *p* < 0.05 were considered significant.

## 3. Results

### 3.1. Time Course of Cytokines Expression

**Figure 1:**
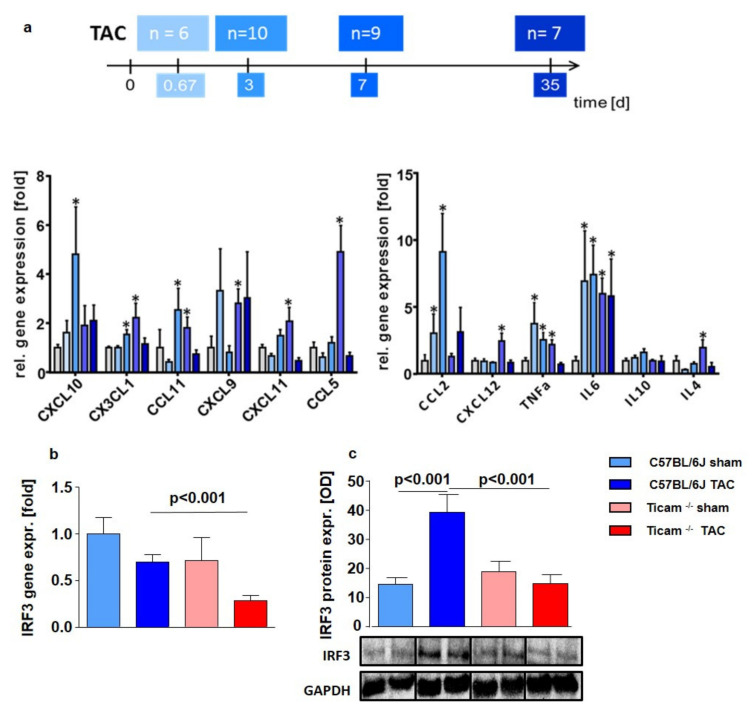
(**a**) Maximum gene expression of cytokines 16 h and d 3 after TAC. Relative mRNA Expression in heart tissue of C57BL/6 mice 16 h, d 3, d 7, and d 35 after TAC (*n* = 6–10; * = *p* < 0.05) compared to the mean of Sham-operated mice. Downstream IRF3 mRNA (**b**) and protein expression (**c**) in heart tissue d7 after TAC, ctrl. vs. TAC (*n* = 5–8).

The maximum (five- to nine-fold) cytokine mRNA expression of the cytokines (IL4, IL6, IL10, CCL2, CCL5, CCL11, CXCL9, CXCL10, CXCL11, CXCL12, CX3CL1, and TNF alpha) was found at 3 and 7 days following TAC in the left ventricular (LV) tissue (Figure 1a).

The downstream target IRF3 showed a similar mRNA expression pattern in the control and the TRIF Group (WT Sham = 1.00 ± 0.58; WT TAC = 0.69 ± 0.14; *p* = n.s.; TRIF^−/−^ Sham = 0.71 ± 0.24; TRIF^−/−^ TAC = 0.33 ± 0.18; *p* = n.s.) (Figure 1b). The difference between the WT and TRIF^−/−^ mice was evident at the protein level: IRF3 protein expression increased 2.7-fold after TAC in WT, while no change was detected in the TRIF^−/−^ mice (WT Sham = 1.00 ± 0.17; WT TAC = 2.72 ± 0.42; *p* < 0.001; TRIF^−/−^ Sham = 0.39 ± 0.11; TRIF^−/−^ TAC = 0.51 ± 0.12; *p* = n.s.; *p* = TAC vs. TAC < 0.01) (Figure 1c).

### 3.2. TRIF-Dependent Cytokine Expression in Heart and LPS Stimulated Monocytes

**Figure 2:**
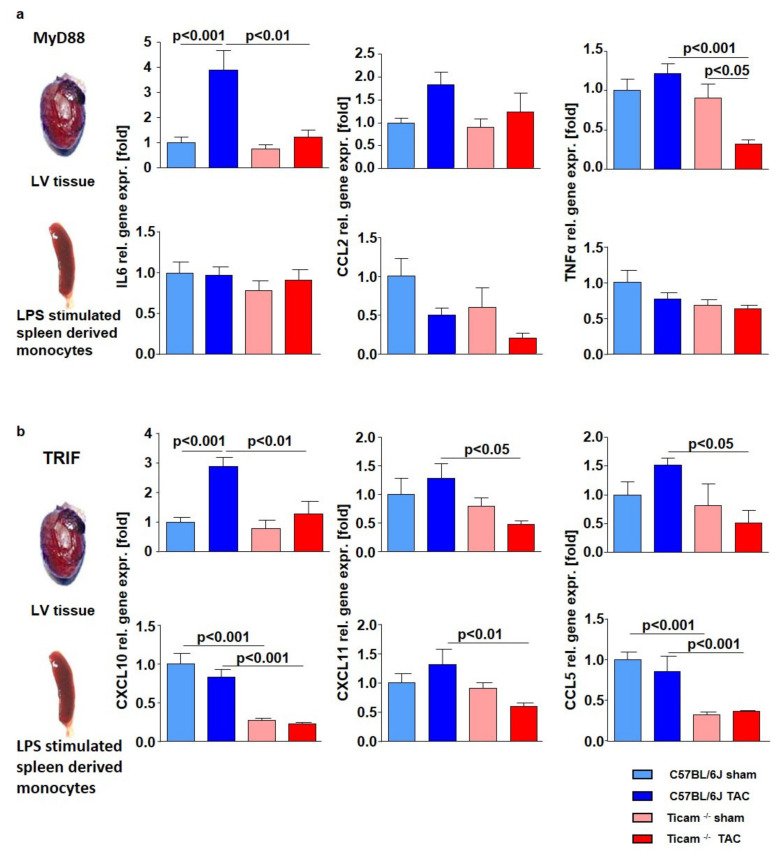
Lack of increase in MyD88 (**a**) and TRIF (**b**) dependent cytokines in TRIF^−/−^mice. Relative mRNA expression in heart tissue and LPS stimulated spleen derived monocytes d 7 after TAC, ctrl. vs. TAC (*n* = 5–8).

Corresponding to the time course analysis, the IL6, CCL2, and TNFα expression increased in the mouse heart tissues. The expression level was similar to the sham-operated WT and TRIF^−/−^ mice. However, following TAC, the gene expression was clearly attenuated in the TRIF^−/−^ mice (Figure 2a). In the LPS-stimulated monocytes of the spleen, the effects were moderate (Figure 2a). As expected, the mRNA expressions of the TRIF-associated cytokines, such as CXCL10, CXCL11, and CCL5, were significantly reduced in the TRIF^−/−^ mice after TAC, but in the WT mice, the cytokine expression increased (Figure 2b). The reduced TRIF-dependent cytokine expression was numerical, albeit not statistically, significant in the LPS- stimulated monocytes of the spleen (Figure 2b).

### 3.3. Echocardiography

Echocardiography was performed on days 0, 7, and 35, respectively. The absence of TRIF tended to slow the development of myocardial hypertrophy in the early stage.

**Figure 3:**
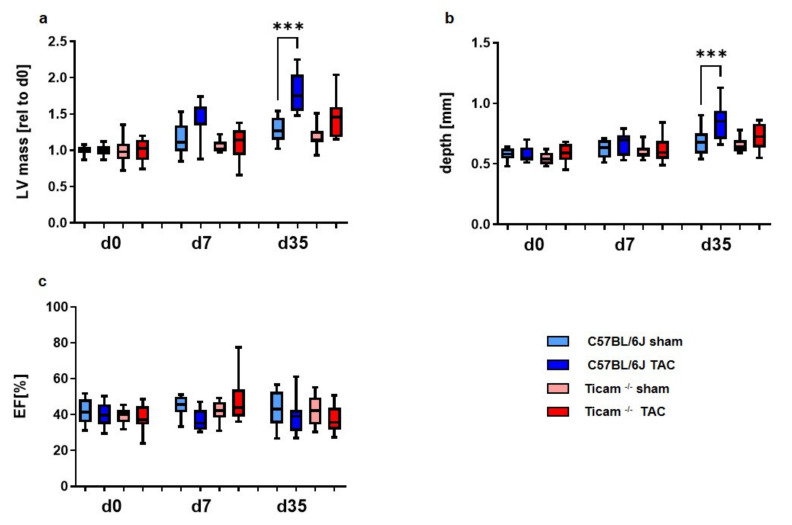
Echocardiographic data at the early (d7) and late stage (d35): (**a**) left ventricular mass, (**b**) depth of the left posterior wall and (**c**) ejection fraction (*n* = 8–10, *** = *p* < 0.001).

Hypertrophic remodeling was initiated much earlier in wild-type mice than in the TRIF-deficient TAC mice (Table 1, Figure 3). In the wild-type animals, the left ventricular posterior wall (LVPW; d), as well as the left ventricular mass (LVM), increased early (d7) compared to the TRIF^−/−^ mice.

In the TRIF^−/−^ mice, the thickening of the left ventricular posterior wall (LVPW; d) and ventricle occurred later at day 35. Similarly, the reduction in the ejection fraction (EF [%]) was delayed in the TRIF^−/−^ mice.

### 3.4. Working Heart Preparation

**Figure 4:**
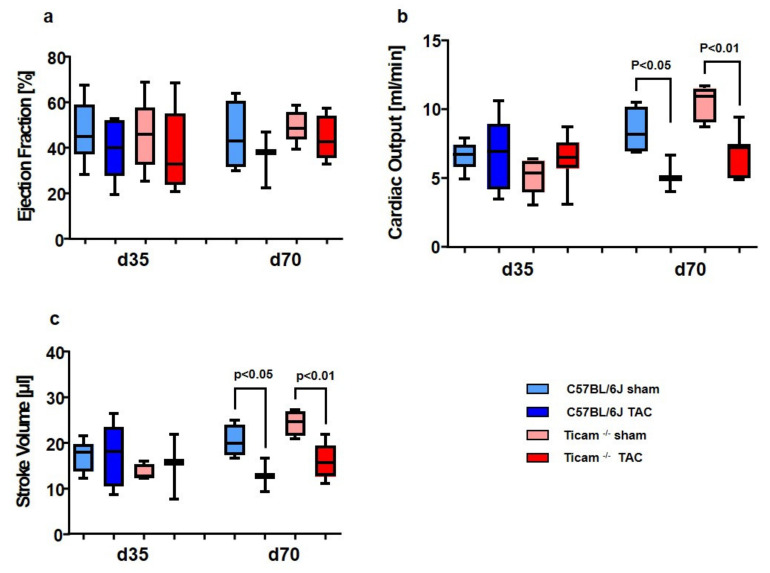
Working heart data showed slightly physiological effects on d 70: (**a**) Ejection fraction, (**b**) cardiac output and (**c**) stroke volume fraction (*n* = 6–9).

As an incidental finding, the hearts of the C57BL/6J animals showed a much more frequent adhesion of the surgical suture to the frontal chest wall than the TRIF^−/−^mice. The functionality of the hearts was examined on day 35 and day 70 with the Langendorff perfusion method in the working heart mode. A moderate reduction in the ejection fraction (Figure 4a) in the TAC mice was observed without a difference between the wild-type and TRIF^−/−^. The slight modulation of hemodynamic effects was also seen in cardiac output (CO, Figure 4b)) and the associated stroke volume (SV, Figure 4c)). These modulations were not visible in the 35-day animals but were pronounced in the 70-day animals. In conclusion, neither the cardiac output nor stroke volume was different between the two groups of animals (Table 2).

### 3.5. Cardiomyocyte Hypertrophy

**Figure 5:**
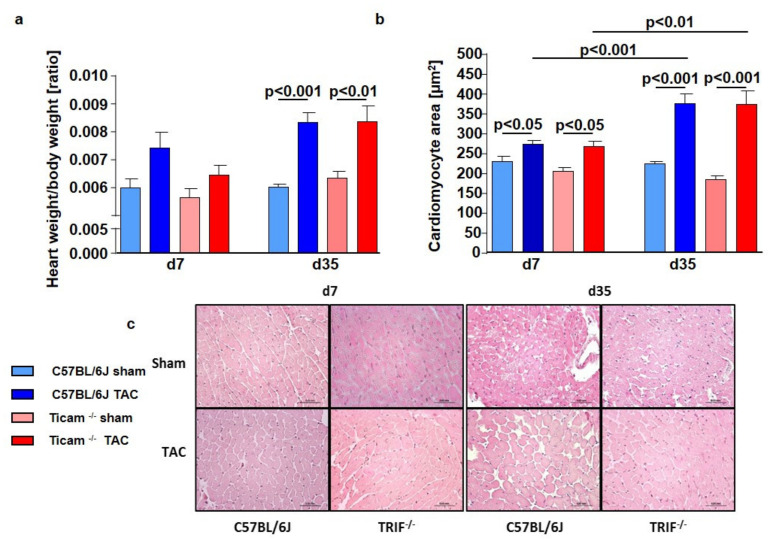
(**a**): Heart weight/body weight ratio of WT and TRIF^−/−^ mice on day 7 and day 35 after TAC, ctrl. vs. TAC (*n* = 5–8). (**b**): Cardiomyocyte area of WT and TRIF^−/−^mice on day 7 and day 35 after TAC, ctrl. vs. TAC (*n* = 6–8). (**c**): Hematoxylin and eosin staining of WT and TRIF^−/−^ derived cardiomyocytes d7 and d35 after TAC, ctrl. vs. TAC (*n* = 6–8).

The TAC-operated animals showed an increase in the heart weight/body weight ratio of approximately 25% versus Sham on day 7 (WT Sham = 5.97 ± 0.34; WT TAC = 7.40 ± 0.56; *p* < 0.01; TRIF^−/−^ Sham = 5.63 ± 0.30; TRIF^−/−^ TAC = 6.46 ± 0.33; *p* < 0.01) and 50% on day 35 (WT Sham = 5.97 ± 0.04; WT TAC = 8.29 ± 0.42, *p* < 0.01; TRIF^−/−^ Sham = 6.32 ± 0.23; TRIF^−/−^ TAC = 8.29 ± 0.58, *p* < 0.01). The increase was similar in both groups (Figure 5a). The HE staining of the cardiomyocytes revealed concordant results (Figure 5c). The cellular size in both groups increased in nearly the same matter: 20% on day 7 (WT Sham = 115.2 ± 6.3 µm^2^; WT TAC = 137.3 ± 4.0 µm^2^; *p* < 0.01; TRIF^−/−^ Sham = 102.9 ± 4.4 µm^2^; TRIF^−/−^ TAC = 134.1 ± 7, µm^2^, *p* < 0.001) and 60% at d35 (WT Sham = 110.2 ± 5.2 µm^2^; WT TAC = 188.6 ± 11.9 µm^2^; *p* < 0.01; TRIF^−/−^ Sham = 99.8 ± 4.0 µm^2^; TRIF^−/−^ TAC = 186.9 ± 17.1 µm^2^; *p* < 0.001) (Figure 5b).

### 3.6. Accumulation of Inflammatory Cell

**Figure 6:**
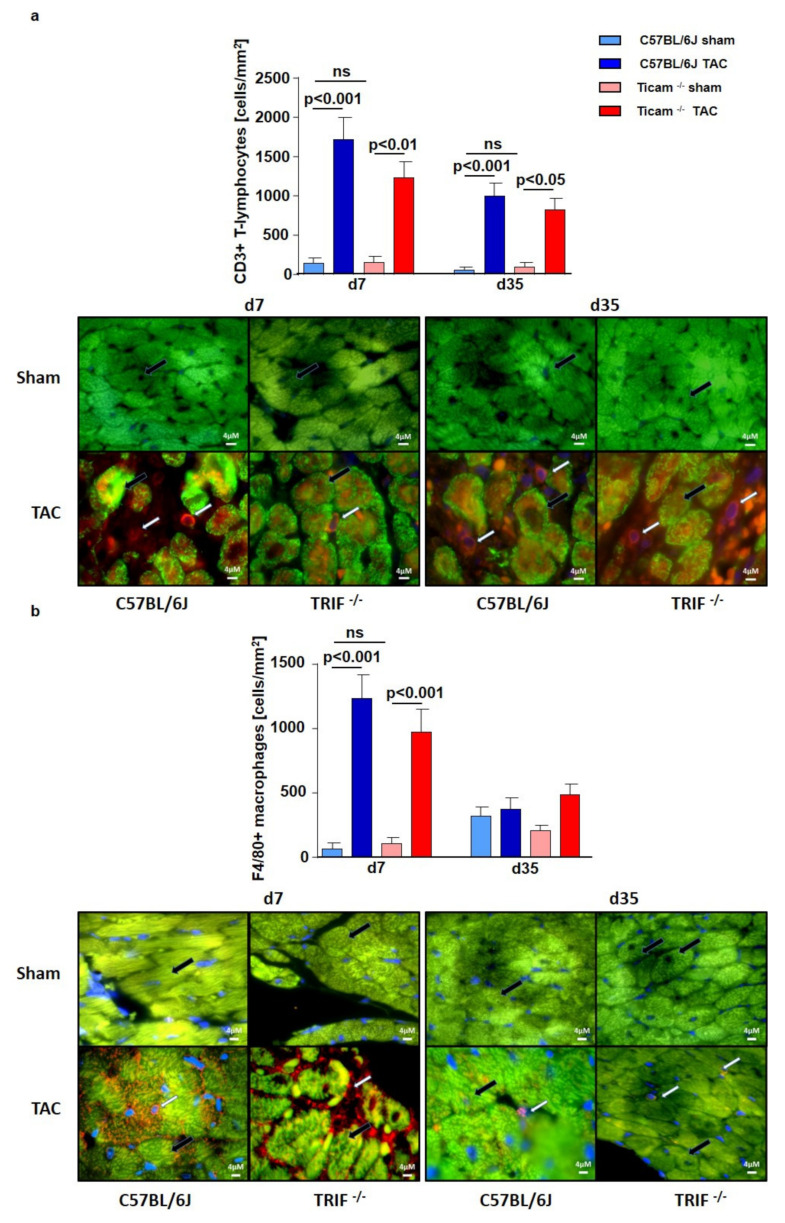
(**a**) IHC staining of CD3 in heart tissue d7 and d35 after TAC, ctrl. vs. TAC (*n* = 3–7). (**b**) IHC staining of F4/80 in heart tissue d7 and d35 after TAC, ctrl. vs. TAC (*n* = 3–9). White arrow: CD3, respectively, F4/80-positive cells, black arrow cardiomyocyte. (ns = not significan).

Immunohistochemical staining with T-lymphocyte specific CD3-antibody showed an increased number of T-cells in both groups, especially on day 7 after TAC. The cell count of the CD3+ T-lymphocytes per mm^2^ was 12-fold higher in the WT group but only eight-fold higher in the TRIF^−/−^ group after TAC compared to the Sham-operated animals (WT Sham = 43.2 ± 70.3 cells/mm^2^; WT TAC = 1718.0 ± 287.9 cells/mm^2^; *p* < 0.001; TRIF^−/−^ Sham = 151.9 ± 74.4 cells/mm^2^; TRIF^−/−^ TAC = 1237.0 ± 202.9 cells/mm^2^; *p* < 0.01, p TAC vs. TAC < 0.05). On day 35, the number of inflammatory T-cells per mm^2^ was lowered and the difference between the Sham- and TAC-operated animals of each mouse strain was similar (WT Sham = 52.3 ± 35.73 cells/mm^2^; WT TAC = 992.5 ± 175.9 cells/mm^2^; *p* < 0.001; TRIF^−/−^ Sham = 93.04 ± 55.2 cells/mm^2^; TRIF^−/−^ TAC = 821.0 ± 143.6 cells/mm^2^; *p* < 0.01) (Figure 6a). The macrophages were stained for F4/80-positive cells. The TAC-operated animals had a significantly higher number of F4/80-positive macrophages per mm^2^ than the Sham-operated animals. Macrophage occurrence was 19-fold higher in the WT mice but only nine-fold higher in TRIF^−/−^ (WT Sham = 65.1 ± 47.9 cells/mm^2^; WT TAC = 1237.0 ± 181.5 cells/mm^2^; *p* < 0.001; TRIF^−/−^ Sham = 108.5 ± 45.1 cells/mm^2^; TRIF^−/−^ TAC = 976.3 ± 172.8 cells/mm^2^; *p* < 0.01). On day 35, the amount in the heart tissue was nearly identical in the TAC- and Sham-operated mice (WT Sham = 320.6 ± 69.7 cells/mm^2^; WT TAC = 376.1 ± 86.5 cells/mm^2^; *p* = n.s.; TRIF^−/−^ Sham = 207.8 ± 43.2 cells/mm^2^; TRIF^−/−^ TAC = 491.2 ± 82.2 cells/mm^2^; *p* = n.s. (Figure 6b).

### 3.7. Fibrosis and TGFβ Signal Pathway

**Figure 7:**
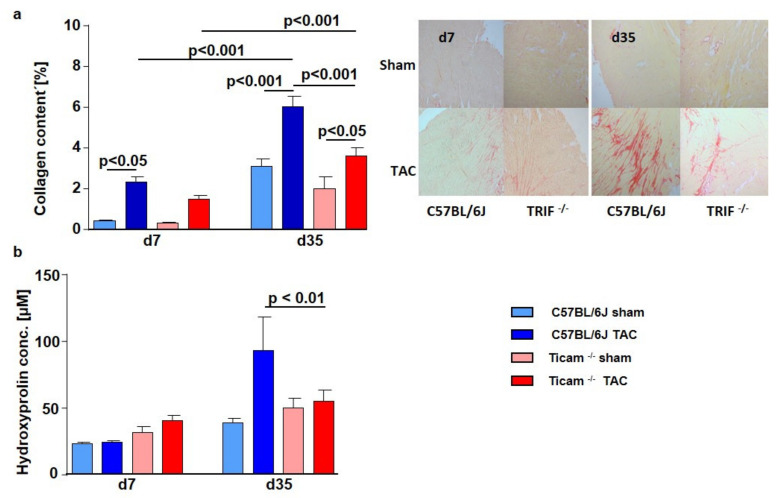
(**a**) Picrosirius Red staining of heart tissue d 7 and d 35 after TAC, ctrl. vs. TAC (*n* = 5–7). (**b**) Hydroxyproline concentration of heart tissue d 7 and d 35 after TAC, ctrl. vs. TAC (*n* = 6–11).

**Figure 8:**
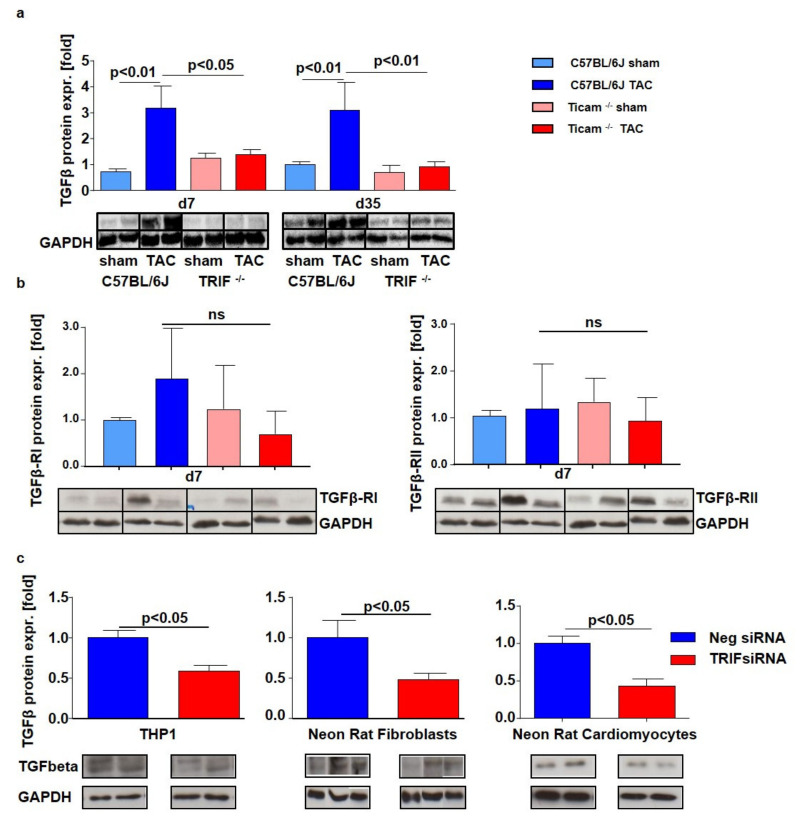
(**a**) Western Blot analysis of TGFβ d 7 and d 35 after TAC, ctrl. vs. TAC (*n* = 5–8). (**b**) Western Blot analysis of TGFβ-RI and RII d 7 after TAC, ctrl. vs. TAC (*n* = 5–8). (**c**) Western Blot analysis of TGFβ in siRNA transfected cells, negative siRNA vs. TRIF siRNA (*n* = 4).

On day 7 after TAC, Picrosirius Red staining of the heart tissue revealed a significant increase in collagen content in the WT mice (WT Sham = 0.41 ± 0.05%, WT TAC = 2.33 ± 0.26%; *p* < 0.05), while in the TRIF^−/−^ mice, the collagen content was attenuated (TRIF^−/−^ Sham = 0.32 ± 0.04%, TRIF^−/−^ TAC = 1.46 ± 0.20%; *p* = n.s): The difference between the two groups was considerably pronounced on day 35. The TRIF^−/−^ mice showed less collagen content in the heart tissue than the WT mice (WT Sham = 3.10 ± 0.38%, WT TAC = 6.02 ± 0.52%; *p* < 0.001; TRIF^−/−^ Sham = 1.99 ± 0.61%, TRIF^−/−^ TAC = 3.62 ± 0.39%; *p* < 0.05) (Figure 7a). Similarly, the hydroxyproline concentration was significantly higher in the WT mice on day 35 after TAC than in TRIF^−/−^ (WT Sham = 37.1 ± 2.3 µM, WT TAC = 93.4 ± 16.9 µM; *p* < 0.001; TRIF^−/−^ Sham = 50.3 ± 4.7 µM, TRIF^−/−^ TAC = 54.8 ± 6.1 µM; *p* = n.s.; p TAC vs. TAC < 0.01) (Figure 7b). Concerning the increased collagen content, the TGFβ protein expression was attenuated in the TRIF^−/−^ heart tissue of the TAC-operated mice on day 7 (WT Sham = 1.00 ± 0.29, WT TAC = 3.17 ± 0.85; *p* < 0.01; TRIF^−/−^ Sham = 1.23 ± 0.19, TRIF^−/−^ TAC = 1.38 ± 0.19; *p* = n.s.; p TAC vs. TAC < 0.05) and at d35 (WT Sham = 1.00 ± 0.11; WT TAC = 3.10 ± 1.07; *p* < 0.01; TRIF^−/−^ Sham = 0.69 ± 0.28; TRIF^−/−^ TAC = 0.91 ± 0.21; *p* = n.s.; p TAC vs. TAC < 0.01) (Figure 8a). In addition, TGFβ receptor protein expression (RI, RII) increased in the control group after TAC, while in the TRIF^−/−^ mice, it remained at the level of the Sham-operated animals (TGFβ-RI: WT Sham = 1.00 ± 0.01, WT TAC = 1.88 ± 2.43; *p* = n.s; TRIF^−/−^ Sham = 1.37 ± 1.00, TRIF^−/−^ TAC = 0.65 ± 1.11; *p* = n.s.; p TAC vs. TAC = n.s.; TGFβ-RII: WT Sham = 1.00 ± 0.12; WT TAC = 1.20 ± 1.03; *p* = n.s.; TRIF^−/−^ Sham = 1.13 ± 0.66; TRIF^−/−^ TAC = 0.84 ± 0.60; *p* = n.s.; p TAC vs. TAC *p* = n.s.) (Figure 8b).

The Western blot analysis of the TRIF-si-transfected THP1 cells (neg-siRNA TX = 1.00 ± 0.09; TRIF-siRNA TX = 0.5 ± 0.07; *p* < 0.05), neonatal rat fibroblasts (neg-siRNA TX = 1.00 ± 0.22; TRIF-siRNA TX = 0.49 ± 0.08; *p* < 0.05), and neonatal rat cardiomyocytes (neg-siRNA TX = 1.00 ± 0.1; TRIF-siRNA TX = 0.43 ± 0.09; *p* < 0.05), showed a remarkably lower TGFβ protein expression in all cell types (Figure 8c).

### 3.8. Fibrosis Associated miRNAs

**Figure 9:**
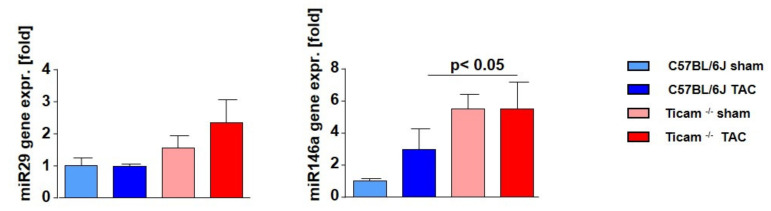
Relative microRNA expression in heart tissue d7 after TAC, ctrl. vs. TAC (*n* = 6–8).

One of the key regulators for the modulation of cardiac fibrosis in the framework of TGFβ signaling is antifibrotic miR-29. On day 7 after TAC, no changes in the miR-29 expression was measurable in the WT mice, while in the TRIF^−/−^ mice, more miR-29 was detected. However, comparing the two TAC groups, there was only a nominal albeit but no significant difference detectable (WT Sham = 1.00 ± 0.25; WT TAC = 0.98 ± 0.08; *p* = n.s.; TRIF^−/−^ Sham = 1.54 ± 0.40; TRIF^−/−^ TAC = 2.34 ± 0.71; *p* = n.s.). miR-146, which represses SMAD4, showed a higher expression level in the Sham-operated TRIF ^−/−^ mice than in WT. After TAC, the miR-146 expression increased in both groups, but the relative expression of miR-146 remained significantly higher in the TRIF^−/−^group (WT Sham = 1.00 ± 0.17; WT TAC = 2.95 ± 1.29; *p* < 0.01; TRIF^−/−^ Sham = 5.51 ± 0.87; TRIF^−/−^ TAC = 5.51 ± 1.64; *p* = n.s.; p Sham vs. Sham < 0.05). (Figure 9).

### 3.9. Proliferation and Apoptosis

**Figure 10:**
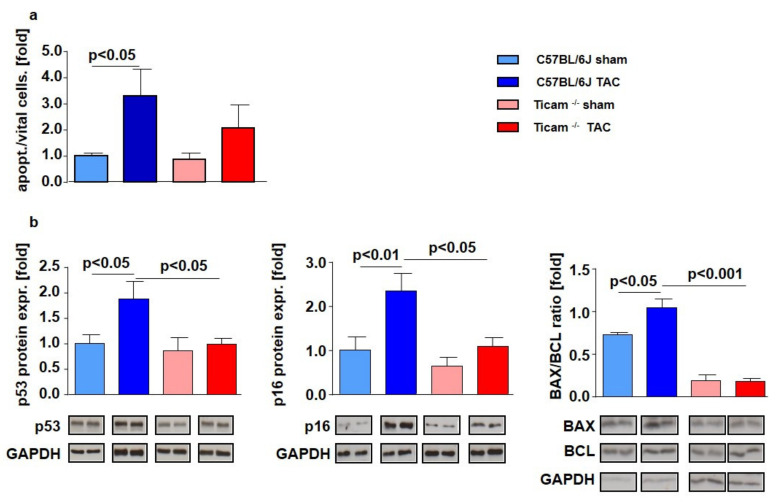
TRIF deficiency leads to less apoptosis and more proliferative capacity of the myocardium and less proliferative capacity in fibroblasts. (**a**) Ratio of vital and apoptotic cells in cross-sections of LV tissue d 7 after TAC, ctrl. vs. TAC (*n* = 8). (**b**) Relative mRNA expression of apoptosis related genes in heart tissue d 7 after TAC, ctrl. vs. TAC (*n* = 5–8).

On day 7, the quantity of apoptotic cells in the myocardium compared to vital cells increased significantly in the WT mice after TAC (WT Sham = 1.00 ± 0.11; WT TAC = 3.295 ± 1.04; *p* < 0.05), while in the TRIF^−/−^ mice, apoptosis was more moderate (TRIF^−/−^ Sham = 1.00 ± 0.23; TRIF^−/−^ TAC = 2.38 ± 0.96; *p* = n.s (Figure 10a).

**Figure 11:**
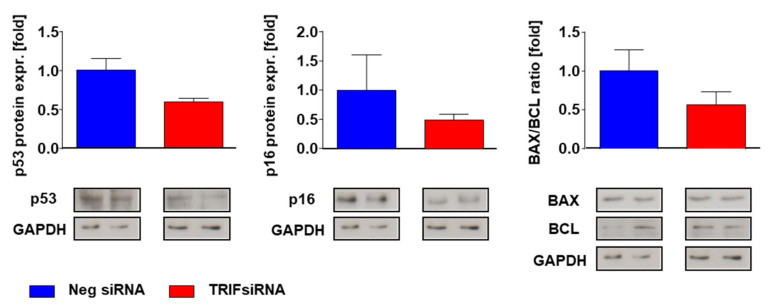
Relative mRNA expression of apoptosis related genes in vitro, neonatal rat cardiomyocytes, negative siRNA vs. TRIF siRNA (*n* = 4).

Proapoptotic p53 protein indicated a two-fold increase in the TAC-operated WT mice, while in the TRIF^−/−^ mice, both groups had the same expression level in the heart tissue (WT Sham = 1.00 ± 0.17; WT TAC = 1.87 ± 0.35; *p* < 0.05; TRIF^−/−^ Sham = 0.86 ± 0.26; TRIF^−/−^ TAC = 0.99 ± 0.11; *p* = n.s.; p TAC vs. TAC < 0.05). Either the p16 protein expression increased only in the control mice after TAC (WT Sham = 1.0 ± 0.30; WT TAC = 2.33 ± 0.40; *p* < 0.01; TRIF^−/−^ Sham = 0.63 ± 0.20; TRIF^−/−^ TAC = 1.08 ± 0.20; *p* = n.s.; p TAC vs. TAC < 0.05). The basic level of the bax/bcl ratio was higher in the C57BL/6 compared to the TRIF^−/−^ mice. After TAC, the bax/bcl ratio increased in the WT but not in the TRIF^−/−^ mice (WT Sham = 1.00 ± 0.04; WT TAC = 1.44 ± 0.13; *p* < 0.05; TRIF^−/−^ Sham = 0.25 ± 0.10; TRIF^−/−^ TAC = 0.24 ± 0.05; *p* = n.s.; p TAC vs. TAC < 0.01) (Figure 10b). In vitro experiments demonstrate that in the TRIF-siRNA transfected neonatal rat cardiomyocytes, the protein expression of p53 (neg-siRNA TX = 1.00 ± 0.15 TRIF-siRNA TX = 0.60 ± 0.05; *p* = n.s.) and p16 (neg-siRNA TX = 1.00 ± 0.36; TRIF-siRNA TX = 0.50 ± 0.10; *p* = n.s.) was decreased after TRIF knockdown. According to that investigation the bax/bcl ratio (neg-siRNA TX = 1.00 ± 0.27; TRIF-siRNA TX = 0.57 ± 0.17; *p* = n.s.) was attenuated in the TRIF si-transfected cardiomyocytes (Figure 11).

## 4. Discussion

Inflammation plays an important role in the adaptive and maladaptive remodeling of the heart. Left-ventricular overload results in myocardial inflammation with the myocardial overexpression of pro-inflammatory cytokines and leukocyte infiltration into the myocardium. Various observational studies in animals and patients have indicated a relationship between the presence of pro-inflammatory markers and pressure overload-induced hypertrophy and fibrosis [7,8,9]. In our study, we discovered an increase in cytokines in the early stage (d3, d7) after TAC. The MyD88- and TRIF-dependent pathways are involved in inflammatory-induced intracellular signaling. While MyD88 is utilized by all known TLRs except for TLR3, TRIF mediates only TLR3- and TLR4-dependent signal cascades. TLR4 is known as a co-player in cardiac remodeling after myocardial infection and cardiac hypertrophy [15,16,17,21,22,23]. We focused on the role of the TLR3/4-TRIF-dependent pathway in afterload-induced myocardial remodeling.

In our study, while MyD88 knockdown yields reduced cardiac hypertrophy after MI and TAC [24,25], TRIF apparently has little influence on the development of afterload-induced cardiomyocyte hypertrophy. In contrast, TRIF is not involved to the same extent, as shown by our data. This is distinct from other forms of cardiomyopathy, e.g., ischemic cardiomyopathy, apoptosis, and ischemia–reperfusion. On a morphological level, no differences in the cardiomyocyte area and in the heart weight/body weight ratio were found in the TRIF^−/−^ mice compared with control at an early (d7) and late stage (d35) following TAC.

However, TRIF knockout led to the downregulation of IRF3 and reduced the downstream gene expression of CXCL10, CXCL11, and CCL5 in LV tissue and in the LPS-stimulated spleen-derived monocytes. Interestingly, differential gene expression was more pronounced locally in heart tissue, possibly because here, following TAC, TRIF inflammatory signaling is strongly switched on. We observed a decrease in T-Lymphocytes and macrophages in the heart tissue of the TAC-operated TRIF^−/−^ mice in the early stage. These cell types are known as common sources of inflammatory cytokines. The reduced number of these cells could be the cause of the decreased expression of inflammatory cytokines in the heart tissue of the TRIF^−/−^ mice after TAC. During further remodeling, changes in cellular accumulation seemed to vanish, most likely because cellular infiltration is an early process. Because of the role of T-lymphocytes and macrophages in the remodeling processes, other immunomodulatory cell (sub-)types were not analyzed, which is a limitation of the study.

Richards et al. [26] showed that TRIF deficiency leads to a decreased macrophage accumulation in atherosclerotic lesions, which is correlated with a significant decrease in lesion burden. In our model, TRIF deficiency led to a pronounced decrease in fibrosis in LV tissue, shown by the reduced collagen content and lower hydroxyl-proline concentration compared to the controls. Former studies characterized the influence of chemokines on the fibrotic process in different kinds of tissue [27,28,29]. In addition, CXCL10, which promotes liver fibrosis [30], was downregulated in TRIF^−/−^ mice.

Reduced LV fibrosis in the TRIF^−/−^ mice comes along with decreased TGFβ protein expression. Through its pleiotropic effects, TGFβ is a key mediator in the transition from inflammation to fibrosis. It exerts effects on all cell types involved in cardiac injury, repair, and remodeling [31]. TGFβ receptors I and II were not upregulated in the TRIF^−/−^ mice after TAC, which led to less signal transduction in the TGFβ induced pathway. In our in vitro experiments, following TRIF knockdown, the protein expression of TGFβ was downregulated in macrophages, fibroblasts, and cardiomyocytes, suggesting that these cell types are equally involved in TGFβ-mediated fibrosis.

Several studies demonstrated the functional role of microRNAs in cardiac remodeling, particularly during cardiac fibrosis [32,33,34,35,36,37]. Herein, TRIF deficiency was associated with an increase in miRNA 29 expression. Van Rooij et al. [38] showed that the miRNA-29 family directly targets mRNA encoding a multitude of ECM-related proteins involved in fibrosis, including collagen, fibrillin, and elastin. The overexpression of miR-29 in fibroblast reduces the expression of collagen. These data indicate that miR-29 acts as a regulator of cardiac fibrosis [37,39,40]. According to our data, miRNA-29 expression in afterload-treated hearts seems to be TRIF-dependent. Another microRNA involved in immune regulation is miR-146a [41]. He et al. [42] suggested that miR-146a was decreased in liver fibrotic tissues. In LV of TRIF^−/−^ mice, miR-146a was upregulated. Our results emphasized miR-146a as part of the TRIF-regulated TGF-beta signaling pathway.

Cardiac remodeling goes along with altered apoptosis and proliferation. Previous studies on MI showed that TLR3-TRIF signaling contributes to ischemic myocardial injury, most likely by mediating cardiac apoptosis, and plays a lesser role in myocardial inflammation during I/R [10]. We demonstrate a TRIF-dependent increase in apoptosis and the proliferation of cardiomyocytes in afterload-induced hypertrophy. siRNA-transfected neonatal rat cardiomyocytes had an attenuated expression of the pro-apoptotic proteins p53 and p16, as well as a decreased bax/bcl ratio. The LV tissue of the TRIF^−/−^ mice showed similar protein-expression patterns as cell culture experiments. Chen et al. [11] indicated that TRIF-TLR-signaling contributes to ischemic myocardial injury, most likely by mediating cardiac apoptosis. Thus, TRIF signaling is not only involved in the TGFβ-dependent fibrotic pathway but also in cellular viability in afterload-induced LV remodeling.

**Figure 12:**
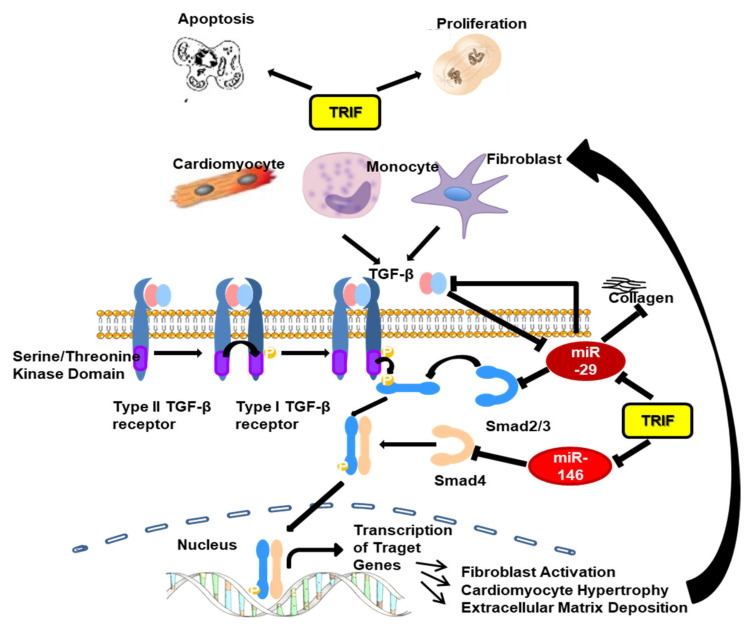
Putative activation of the inflammatory/fibrotic pathway after TAC with focus on the role of TRIF.

In summary, the Toll-like adapter TRIF influences afterload-induced myocardial remodeling at several molecular and cellular sites. In the early stage of afterload-induced remodeling, TRIF is involved in intracellular inflammatory signaling by supporting inflammatory cell (monozyte, CD3 T-cell) -infiltration and modifying inflammatory chemokine gene expression. TRIF signaling enhances apoptosis, attenuates the proliferation of cardiomyocytes, and stimulates the proliferation of fibroblasts. TRIF is involved in the TGFβ-dependent fibrotic pathway. The downregulation of TRIF leads to profoundly reduced fibrosis via TGFβ signaling and the increased expression of anti-fibrotic microRNAs (Figure 12). In summary, TRIF deficiency decelerates the progression of heart failure in the early stage of maladaptive remodeling. During the later time course of maladaptive remodeling, the signaling pathways shift away from TRIF-dependent regulation towards alternative (MyD88-dependent) pathways, diminishing initial molecular differences at the functional level.

## Figures and Tables

**Table 1:** Echocardiographic data of Wild Type and TRIF^−/−^ mice at d7 and d35.

Parameter	WT			TRIF^−/−^		
	Sham	TAC	*p*-Value	Sham	TAC	*p*-Value
**d7: LVPW; d [mm]**	0.64 ± 0.02	0.79 ± 0.06	<0.05	0.64 ± 0.03	0.65 ± 0.04	ns
**d7: LVM [fold]**	1.00 ± 0.04	1.45 ± 0.07	<0.001	1.00 ± 0.02	1.16 ± 0.08	ns
**d35: LVPW; d [mm]**	0.64 ± 0.02	0.74 ± 0.05	<0.001	0.65 ± 0.02	0.73 ± 0.03	<0.001
**d35: LVM [fold]**	1.20 ± 0.05	1.62 ± 0.14	<0.001	1.19 ± 0.05	1.50 ± 0.08	<0.001
**d7: EF [%]**	40.33 ± 1.44	39.34 ± 1.69	ns	42.31 ± 1.51	42.76 ± 1.97	ns
**d35 EF [%]**	40.80 ± 2.59	38.03 ± 1.57	ns	41.54 ± 2.37	36.64 ± 2.42	ns

**Table 2:** Working heart data of Wild Type and TRIF^−/−^ mice at d35 and d70.

Parameter	WT			TRIF^−/−^		
	Sham	TAC	*p*-Value	Sham	TAC	*p*-Value
**d35: EF [%]**	46.74 ± 5.67	39.67 ± 6.05	ns	45.15 ± 7.02	39.03 ± 6.66	ns
**d70: EF [%]**	44.81 ± 7.56	35.66 ± 7.19	ns	49.32 ± 3.12	44.21 ± 4.36	ns
**d35: CO [ml/min]**	6.60 ± 0.44	6.63 ± 1.21	ns	5.15 ± 0.60	6.43 ± 0.69	ns
**d70: CO [ml/min]**	8.43 ± 0.86	5.22 ± 077	ns	10.41 ± 0.57	6.63 ± 0.64	<0.01
**d35: SV [µl]**	16.94 ± 0.56	17.14 ± 3.12	ns	13.34 ± 0.89	14.49 ± 1.56	ns
**d70: SV [µl]**	30.31 ± 1.76	12.79 ± 2.10	<0.05	24.27 ± 1.41	15.81 ± 1.51	<0.001

## Data Availability

Not applicable.

## Supplementary Materials

The following supporting information can be downloaded at: https://www.mdpi.com/article/10.3390/biomedicines10102636/s1; Table S1: Real-Time PCR Primer. Table S2: Antibodies for Immunohistochemistry. Figure S1: Echocardiographic measurements. (a) Overview of a M-mode measurement in the heart of a WT Sham mouse at d35. (b) B-mode recording in parasternal longitudinal axis and corresponding M-mode (pictures b and c kindly provided by S. Puhl). Figure S2: TRIF knockout in TRIF^−/−^ mice.

## References

[1] Dorn G.W., Robbins J., Sugden P.H. (2003). Phenotyping Hypertrophy: Eschew Obfuscation. Circ. Res..

[2] Hunter J.J., Chien K.R. (1999). Signaling pathways for cardiac hypertrophy and failure. N. Engl. J. Med..

[3] Levy D., Garrison R.J., Savage D.D., Kannel W.B., Castelli W.P. (1990). Prognostic implications of echocardiographically determined left ventricular mass in the Framingham Heart Study. N. Engl. J. Med..

[4] Esposito G., Rapacciuolo A., Naga Prasad S.V., Takaoka H., Thomas S.A., Koch W.J., Rockman H.A. (2002). Genetic alterations that inhibit in vivo pressure-overload hypertrophy prevent cardiac dysfunction despite increased wall stress. Circulation.

[5] Diwan A., Dorn G.W. (2007). Decompensation of cardiac hypertrophy: Cellular mechanisms and novel therapeutic targets. Physiology.

[6] Gordon J.W., Shaw J.A., Kirshenbaum L.A. (2011). Multiple Facets of NF-κB in the Heart. Circ. Res..

[7] Mann D.L. (2002). Inflammatory mediators and the failing heart: Past, present, and the foreseeable future. Circ. Res..

[8] Damas J.K., Eiken H.G., Oie E., Bjerkeli V., Yndestad A., Ueland T., Tonnessen T., Geiran O.R., Aass H., Simonsen S. (2000). Myocardial expression of CC- and CXC-chemokines and their receptors in human end-stage heart failure. Cardiovasc. Res..

[9] Gullestad L., Aukrust P. (2005). Review of trials in chronic heart failure showing broad-spectrum anti-inflammatory approaches. Am. J. Cardiol..

[10] Everett B.M., Cornel J., Lainscak M., Anker S.D., Abbate A., Thuren T., Libby P., Glynn R.J., Ridker P.M. (2019). Anti-Inflammatory Therapy with Canakinumab for the Prevention of Hospitalization for Heart Failure. Circulation.

[11] Chen C., Feng Y., Zou L., Wang L., Chen H.H., Cai J.Y., Xu J.M., Sosnovik D.E., Chao W. (2014). Role of extracellular RNA and TLR3-Trif signaling in myocardial ischemia-reperfusion injury. J. Am. Heart Assoc..

[12] Hardarson H.S., Baker J.S., Yang Z., Purevjav E., Huang C.H., Alexopoulou L., Li N., Flavell R.A., Bowles N.E., Vallejo J.G. (2007). Toll-like receptor 3 is an essential component of the innate stress response in virus-induced cardiac injury. American journal of physiology. Heart Circ. Physiol..

[13] Riad A., Westermann D., Zietsch C., Savvatis K., Becher P.M., Bereswill S., Heimesaat M.M., Lettau O., Lassner D., Dorner A. (2011). TRIF is a critical survival factor in viral cardiomyopathy. J. Immunol..

[14] Negishi H., Osawa T., Ogami K., Ouyang X., Sakaguchi S., Koshiba R., Yanai H., Seko Y., Shitara H., Bishop K. (2008). A critical link between Toll-like receptor 3 and type II interferon signaling pathways in antiviral innate immunity. Proc. Natl. Acad. Sci. USA.

[15] Kaczorowski D.J., Nakao A., Vallabhaneni R., Mollen K.P., Sugimoto R., Kohmoto J., Zuckerbraun B.S., McCurry K.R., Billiar T.R. (2009). Mechanisms of Toll-like receptor 4 (TLR4)-mediated inflammation after cold ischemia/reperfusion in the heart. Transplantation.

[16] Li Y.H., Ha T.Z., Chen Q., Li C.F. (2005). Role of MyD88-dependent nuclear factor-kappaB signaling pathway in the development of cardiac hypertrophy in vivo. Zhonghua Yi Xue Za Zhi.

[17] Ha T., Li Y., Hua F., Ma J., Gao X., Kelley J., Zhao A., Haddad G.E., Williams D.L., William Browder I. (2005). Reduced cardiac hypertrophy in toll-like receptor 4-deficient mice following pressure overload. Cardiovasc. Res..

[18] Hoebe K., Du X., Georgel P., Janssen E., Tabeta K., Kim S., Goode J., Lin P., Mann N., Mudd S. (2003). Identification of Lps2 as a key transducer of MyD88-independent TIR signalling. Nature.

[19] Custodis F., Eberl M., Kilter H., Bohm M., Laufs U. (2006). Association of RhoGDIalpha with Rac1 GTPase mediates free radical production during myocardial hypertrophy. Cardiovasc. Res..

[20] Reil J.C., Hohl M., Reil G.H., Granzier H.L., Kratz M.T., Kazakov A., Fries P., Muller A., Lenski M., Custodis F. (2013). Heart rate reduction by If-inhibition improves vascular stiffness and left ventricular systolic and diastolic function in a mouse model of heart failure with preserved ejection fraction. Eur. Heart J..

[21] Kaczorowski D.J., Nakao A., McCurry K.R., Billiar T.R. (2009). Toll-like receptors and myocardial ischemia/reperfusion, inflammation, and injury. Curr. Cardiol. Rev..

[22] Kaczorowski D.J., Tsung A., Billiar T.R. (2009). Innate immune mechanisms in ischemia/reperfusion. Front. Biosci..

[23] Jiang D.S., Zhang X.F., Gao L., Zong J., Zhou H., Liu Y., Zhang Y., Bian Z.Y., Zhu L.H., Fan G.C. (2014). Signal regulatory protein-alpha protects against cardiac hypertrophy via the disruption of toll-like receptor 4 signaling. Hypertension.

[24] Singh M.V., Swaminathan P.D., Luczak E.D., Kutschke W., Weiss R.M., Anderson M.E. (2012). MyD88 mediated inflammatory signaling leads to CaMKII oxidation, cardiac hypertrophy and death after myocardial infarction. J. Mol. Cell Cardiol..

[25] Ha T., Hua F., Li Y., Ma J., Gao X., Kelley J., Zhao A., Haddad G.E., Williams D.L., Browder I.W. (2006). Blockade of MyD88 attenuates cardiac hypertrophy and decreases cardiac myocyte apoptosis in pressure overload-induced cardiac hypertrophy in vivo. American journal of physiology. Heart Circ. Physiol..

[26] Richards M.R., Black A.S., Bonnet D.J., Barish G.D., Woo C.W., Tabas I., Curtiss L.K., Tobias P.S. (2013). The LPS2 mutation in TRIF is atheroprotective in hyperlipidemic low density lipoprotein receptor knockout mice. Innate Immun..

[27] Smith R.E., Strieter R.M., Zhang K., Phan S.H., Standiford T.J., Lukacs N.W., Kunkel S.L. (1995). A role for C-C chemokines in fibrotic lung disease. J. Leukoc. Biol..

[28] Tesch G.H. (2008). MCP-1/CCL2: A new diagnostic marker and therapeutic target for progressive renal injury in diabetic nephropathy. American journal of physiology. Ren. Physiol..

[29] Kershenobich Stalnikowitz D., Weissbrod A.B. (2003). Liver fibrosis and inflammation. A review. Ann. Hepatol..

[30] Hintermann E., Bayer M., Pfeilschifter J.M., Luster A.D., Christen U. (2010). CXCL10 promotes liver fibrosis by prevention of NK cell mediated hepatic stellate cell inactivation. J. Autoimmun..

[31] Dobaczewski M., Chen W., Frangogiannis N.G. (2011). Transforming growth factor (TGF)-beta signaling in cardiac remodeling. J. Mol. Cell Cardiol..

[32] Wang B., Komers R., Carew R., Winbanks C.E., Xu B., Herman-Edelstein M., Koh P., Thomas M., Jandeleit-Dahm K., Gregorevic P. (2012). Suppression of microRNA-29 Expression by TGF-β1 Promotes Collagen Expression and Renal Fibrosis. J. Am. Soc. Nephrol..

[33] Kriegel A.J., Liu Y., Fang Y., Ding X., Liang M. (2012). The miR-29 family: Genomics, cell biology, and relevance to renal and cardiovascular injury. Physiol. Genom..

[34] Kumarswamy R., Thum T. (2013). Non-coding RNAs in Cardiac Remodeling and Heart Failure. Circ. Res..

[35] Jayawardena T.M., Egemnazarov B., Finch E.A., Zhang L., Payne J.A., Pandya K., Zhang Z., Rosenberg P., Mirotsou M., Dzau V.J. (2012). MicroRNA-Mediated In Vitro and In Vivo Direct Reprogramming of Cardiac Fibroblasts to Cardiomyocytes. Circ. Res..

[36] Schroen B., Heymans S. (2011). Small but smart—microRNAs in the center of inflammatory processes during cardiovascular diseases, the metabolic syndrome and aging. Cardiovasc. Res..

[37] Creemers E.E., Pinto Y.M. (2011). Molecular mechanisms that control interstitial fibrosis in the pressure-overloaded heart. Cardiovasc. Res..

[38] Van Rooij E., Sutherland L.B., Thatcher J.E., DiMaio J.M., Naseem R.H., Marshall W.S., Hill J.A., Olson E.N. (2008). Dysregulation of microRNAs after myocardial infarction reveals a role of miR-29 in cardiac fibrosis. Proc. Natl. Acad. Sci. USA.

[39] Lan H.Y. (2011). Diverse roles of TGF-beta/Smads in renal fibrosis and inflammation. Int. J. Biol. Sci..

[40] Heggermont W.A., Heymans S. (2012). MicroRNAs are involved in end-organ damage during hypertension. Hypertension.

[41] Labbaye C., Testa U. (2012). The emerging role of MIR-146A in the control of hematopoiesis, immune function and cancer. J. Hematol. Oncol..

[42] He Y., Huang C., Sun X., Long X.R., Lv X.W., Li J. (2012). MicroRNA-146a modulates TGF-beta1-induced hepatic stellate cell proliferation by targeting SMAD4. Cell Signal.

