# Supplementary material for: Inhibition of TRIF-Dependent Inflammation Decelerates Afterload-Induced Myocardial Remodeling
Source: Biomedicines. 2022 Oct 19;10(10):2636. doi: 10.3390/biomedicines10102636 (PMC9599817; doi:10.3390/biomedicines10102636)
Supplement: Supplementary file 1 [file biomedicines-10-02636-s001.zip › biomedicines-1893725-supplementary.pdf]

Online Supplement:

Table S1. Real Time PCR Primer.

Target	Forward Primer	Reverse Primer
18s	TCA ACA CGG GAA ACC TCA C	ACC AGA CAA ATC GCT CCA C
CCL2	TGC CCT AAG GTC TTC AGC AC	AAA ATG GAT CCA CAC CTT GC
CCL5	GAC ACC ACA CCC TGC TGC T	TAC TCC TTG ATG TGG GCA CG
CCL11	ACC GAG TGG TTA GCT ACC AGT TG	TGG TGA TGA AGA TGA CCC CTG
CX3CL1	TTA CGG CTA AGC CTC AGA GCA	CTC GGC CAA ACG GTG GT
CXCL9	AAT GCA CGA TGC TCC TGC A	AGG TCT TTG AGG GAT TTG TAG TGG
CXCL10	CCC ACG TGT TGA GAT CAT TG	CAC TGG GTA AAG GGG AGT GA
CXCL11	AAG TCA CGT GCA CAC TCC AC	CGT GTG CCT CGT GAT ATT TG
CXCL12	AAA CCA GTC AGC CTG AGC TAC C	GGC TCT GGC GAT GTG GC
HPRT	CAG GCC AGA CTT TGT TGG AT	CCG CTG TCT TTT AGG CTT TG
IL4	GCA ACG AAG AAC ACC ACA GA	ATC GAA AAG CCC GAA AGA GT
IL6	TCC TAC CCC AAT TTC CAA TG	ACC ACA GTG AGG AAT GTC CA
IL10	GCT CTT ACT GAC TGG CAT GAF	CGC AGC TCT AGG AGC ATG TG
IRF3	ACG TGT CAA CCT GGA AGA GG	GGC ACC CAG ATG TAC GAA GT
TNFα	GAT TAT GGC TCA GGG TCC AA	CTC CCT TTG CAG AAC TCA GG

TRIF	TTG GGA CAC GAT CTA CGA CA	AGG AGC ACA GGA GAA AGC AG

Table S2. Antibodies for Immunhistochemistry.

ANTIGEN	PRIMARY ANTIBODY	DILUTION
**CD3**	Polyclonal anti-CD3 IgG, Rabbit<br>(abcam, Cambridge, UK)	1:30
**F4/80**	Monoclonal anti-F4/80 IgG, Rat<br>(abcam, Cambridge, UK)	1:30
**A-SARCOMERIC ACTIN**	Monoclonal anti-α-sarcomeric Actin IgG, Mouse, (Dianova, Hamburg)	1:30
**SPECIES**	SECONDARY ANTIBODY	
**MOUSE**	FITC-conjugated anti-mouse IgM<br>(Dianova, Hamburg)	1:50
**GOAT**	TRITC-conjugated anti-goat IgG<br>(Dianova, Hamburg)	1:30
**RAT**	Biotin-conjugated anti-rat IgG<br>(Dianova, Hamburg)	1:30
**RABBIT**	Biotin-conjugated anti-rabbit IgG<br>(Dianova, Hamburg)	1:30
	Streptavidin-TRITC (Dianova, Hamburg)	1:50

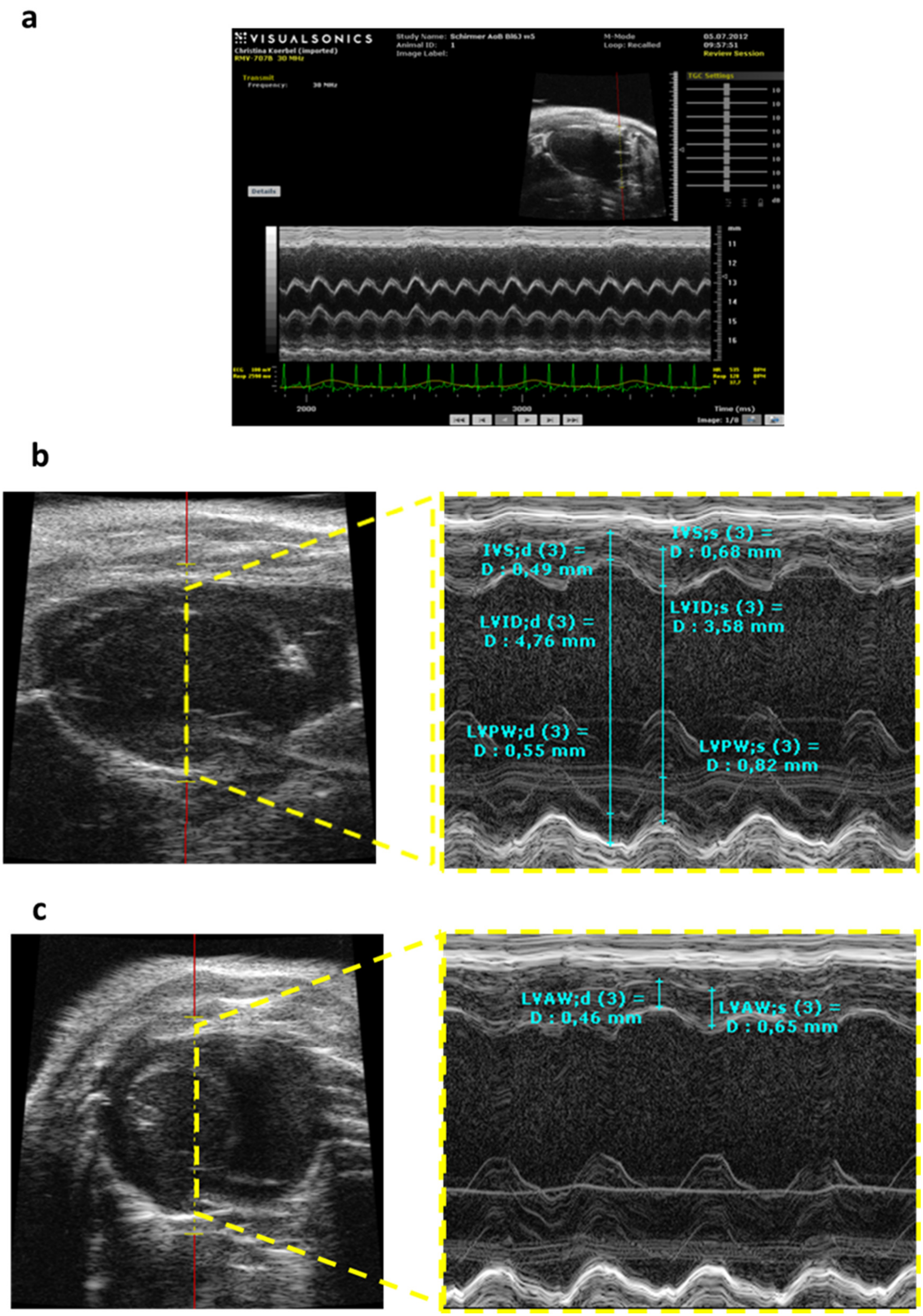

Figure S1. Echocardiographic measurements. (**a**) Overview of a M-mode measurement in the heart of a WT Sham mouse at d35. (**b**) B-mode recording in parasternal longitudinal axis und corresponding M-mode (pictures (**b**) and (**c**) kindly provided from S. Puhl).

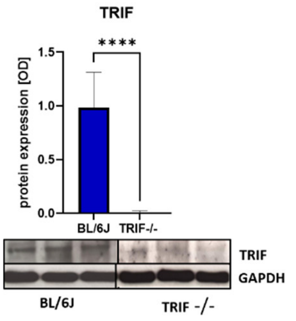

Figure S2. TRIF knock out in TRIF -/- mice.
